# Effect of transcatheter edge-to-edge repair device position on diastolic hemodynamic parameters: An echocardiography-based simulation study

**DOI:** 10.3389/fcvm.2022.915074

**Published:** 2022-08-24

**Authors:** Katharina Vellguth, Fabian Barbieri, Markus Reinthaler, Mario Kasner, Ulf Landmesser, Titus Kuehne, Anja Hennemuth, Lars Walczak, Leonid Goubergrits

**Affiliations:** ^1^Institute of Computer-Assisted Cardiovascular Medicine, Charité—Universitätsmedizin Berlin, Corporate Member of Freie Universität Berlin and Humboldt-Universität zu Berlin, Berlin, Germany; ^2^Department of Cardiology, Charité—Universitätsmedizin Berlin, Corporate Member of Freie Universität Berlin and Humboldt-Universität zu Berlin, Berlin, Germany; ^3^Institute of Active Polymers and Berlin-Brandenburg Center for Regenerative Therapies, Helmholtz-Zentrum Hereon, Teltow, Germany; ^4^German Center for Cardiovascular Research (DZHK), Berlin, Germany; ^5^Berlin Institute of Health at Charité—Universitätsmedizin Berlin, Berlin, Germany; ^6^Deutsches Herzzentrum der Charité—Medical Heart Center of Charité and German Heart Institute Berlin, Berlin, Germany; ^7^Fraunhofer MEVIS, Bremen, Germany; ^8^Einstein Center Digital Future, Berlin, Germany

**Keywords:** mitral valve, mitral regurgitation, transcathether edge-to-edge repair, iatrogenic mitral stenosis, patient-specific, therapy planning, computational fluid dynamics

## Abstract

**Background:**

Transcatheter edge-to-edge repair (TEER) has developed from innovative technology to an established treatment strategy of mitral regurgitation (MR). The risk of iatrogenic mitral stenosis after TEER is, however, a critical factor in the conflict of interest between maximal reduction of MR and minimal impairment of left ventricular filling. We aim to investigate systematically the impact of device position on the post treatment hemodynamic outcome by involving the patient-specific segmentation of the diseased mitral valve.

**Materials and methods:**

Transesophageal echocardiographic image data of ten patients with severe MR (age: 57 ± 8 years, 20% female) were segmented and virtually treated with TEER at three positions by using a position based dynamics approach. Pre- and post-interventional patient geometries were preprocessed for computational fluid dynamics (CFD) and simulated at peak-diastole with patient-specific blood flow boundary conditions. Simulations were performed with boundary conditions mimicking rest and stress. The simulation results were compared with clinical data acquired for a cohort of 21 symptomatic MR patients (age: 79 ± 6 years, 43% female) treated with TEER.

**Results:**

Virtual TEER reduces the mitral valve area (MVA) from 7.5 ± 1.6 to 2.6 ± 0.6 cm^2^. Central device positioning resulted in a 14% smaller MVA than eccentric device positions. Furthermore, residual MVA is better predictable for central than for eccentric device positions (*R*^2^ = 0.81 vs. *R*^2^ = 0.49). The MVA reduction led to significantly higher maximal diastolic velocities (pre: 0.9 ± 0.2 m/s, post: 2.0 ± 0.5 m/s) and pressure gradients (pre: 1.5 ± 0.6 mmHg, post: 16.3 ± 9 mmHg) in spite of a mean flow rate reduction by 23% due to reduced MR after the treatment. On average, velocities were 12% and pressure gradients were 25% higher with devices in central compared to lateral or medial positions.

**Conclusion:**

Virtual TEER treatment combined with CFD is a promising tool for predicting individual morphometric and hemodynamic outcomes. Such a tool can potentially be used to support clinical decision making, procedure planning, and risk estimation to prevent post-procedural iatrogenic mitral stenosis.

## 1. Introduction

Mitral regurgitation (MR) is one of the leading acquired valvular heart diseases in western societies with an increasing prevalence in people over 65 years of age (1, 2). The overall number of cases will rise further with increasing life expectancy and a growing population. While the gold standard for therapy is still found in surgical mitral valve repair, patients with high or prohibitive surgical risk may also be treated by transcatheter edge-to-edge repair (TEER) (3). The general principle of this treatment is to permanently connect the anterior and posterior leaflet at their tips. In case of a primary MR, e.g., due to a prolapse or flail leaflet, the device is supposed to catch the failing part of the leaflet and hold it back in position during systole. In the case of secondary MR, TEER is slightly narrowing the mitral annulus by applying a strain on the mitral leaflets, pulling them toward the orifice center and obtaining an improved coaptation.

The technique has first been proposed about 30 years ago by the Italian surgeon Ottavio Alfieri (4) as the Alfieri-stitch, performed as open heart surgery, and has resulted in two device series for TEER therapy to date. The MitraClip™(Abbott Laboratories, Abbot Park, IL, USA) was the first CE marked TEER device to be certified in 2013 and was investigated broadly in the two EVEREST studies (5, 6). In 2019, the PASCAL device (Edwards Lifesciences, Irvine, CA, USA) received a CE mark as the second device system on the market. Up to now, there are several generations and sizes of each device available (7). Comparison in the literature between MitraClip™ and PASCAL regarding clinical usage aligns with our own experience: a higher flexibility in adaptation to patient specific valve characteristics is reached with PASCAL, while the MitraClip™ system is more likely to allow shorter intervention times (7, 8).

However, recurrence of MR and the risk of iatrogenic mitral stenosis (MS) are major issues and reduce the therapeutic effect (9–12). The opinions on residual and recurrent MR are rather concordant (13, 14), whereas the risk of iatrogenic MS is discussed controversially. Early studies and case studies did not find evidence of an increased risk of post-op stenosis (5, 6, 15), while more recent studies witnessed cases of the high mitral gradient at diastolic filling after TEER in spite of seemingly normal pressure gradients during the intervention (9, 16). The challenge of balancing between residual regurgitation and increased mitral pressure gradient (MPG) after TEER is mentioned by several researchers (11, 14, 17, 18). Singh et al. (19) state an overall underestimated risk of iatrogenic MS and further call the best choice for measuring the MPG an “unanswered question.” An algorithm for estimating the required pre-interventional mitral valve area (MVA) to avoid an iatrogenic MS was developed by Kassar et al. (20). It is based on 3D ultrasound data and takes into account the amount of TEER devices and their position.

TEER procedures, in contrast to surgical interventions, are performed on the beating heart and thus allow for real-time monitoring of hemodynamic parameters, such as left atrial pressure, residual regurgitation, and MPG. Since the hemodynamic characteristics under anesthesia or sedation might not be comparable to hemodynamics in an awake state or even under physical stress, drug-induced stress testing represents a valuable option to test hemodynamics with a TEER device in place. This is, however, only done if necessary in cases of very low stroke volume (21) and not recommended to be used routinely as it bears potential side effects.

A clinical routine of TEER interventions lacks planning tools for device placing and risk assessment, particularly for borderline cases. Planning tools should ideally not only take individual patient characteristics into account but be also able to predict post-interventional residual MVA and MPGs under different activity levels to estimate the risk of MS.

Computational methods and image-based modeling provide tools to investigate several hemodynamic parameters on the basis of patient specific input data. Such approaches have, for instance, been applied to investigate left ventricular hemodynamic flow structures (22–24), the outcome after implantation of biological and mechanical aortic valve prostheses (25), MV tissue properties (26), as well as mitral hemodynamics with and without simulation of diseases and treatment (27). Caballero et al. (18) and Errthum et al. (28) were the first to use advanced computational methods to systematically investigate post-interventional hemodynamic characteristics for one case and several TEER strategies, as well as for specific devices. Lately, Dabiri et al. (29) analyzed a bigger cohort with regard to residual MR by means of finite element modeling and smoothed particle hydrodynamics.

In this work, we want to investigate the influence of the device position on diastolic hemodynamic parameters with regard to iatrogenic stenosis at an individual level of treatment planning. Therefore, we systematically apply virtual TEER treatment in a cohort of 10 patients at three different positions with position based dynamics. Diastolic hemodynamic parameters at conditions of rest and moderate stress are subsequently simulated by means of a low-complexity computational fluid dynamics (CFD) approach (30). The simulation results are further compared to clinical routine data of mitral TEER patients for a plausibility check.

## 2. Materials and methods

**Figure 1:**
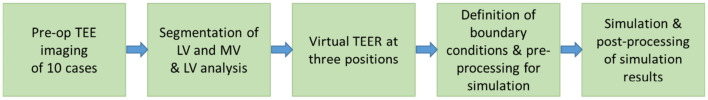
Workflow of this *in silico* study to investigate the effect of trans-catheter edge-to-edge repair (TEER) device position on mitral valve area (MVA) and hemodynamic parameters.

The workflow of this study is displayed in Figure 1. Patient-specific geometries of LV and MV obtained from 3D transesophageal echocardiography (TEE) data are virtually treated by using a position based dynamics approach. Pre- and post-treatment diastolic hemodynamic parameters are further simulated with CFD.

### 2.1. Patient data

A cohort of 10 patients (age: 57±8 years, BSA: 1.96[1.94–2.13], *n* = 2 female, MR grade III, NYHA class III), diagnosed with severe primary mitral regurgitation, was retrospectively analyzed. An aspired share of 50% female cases within the cohort could not be achieved due to a limited database. All patients showed a primary mitral insufficiency without any further heart valve pathology and underwent surgical mitral valve repair since they were not considered high-risk patients. As no suitable 3D TEE data of a patient cohort receiving TEER data was available at the time of this study, we developed this workflow on a cohort with the same pathology but different treatment. Table 1 lists the patient data of the cohort. Written consent was obtained from all of these 10 patients, and the procedures were approved by the local Ethical Committees (Ethikkommission Charité—Universitätsmedizin Berlin: EA2/093/16).

**Table 1:** Clinical and demographic data of study cohort.

**Patient**	**1**	**2**	**3**	**4**	**5**	**6**	**7**	**8**	**9**	**10**	**Mean ±std/Median [IQR]**
Sex	F	M	M	M	M	M	F	M	M	M	–
Age [years]	65	63	64	66	53	65	52	44	60	46	57 ± 8
Body surface area [m^2^]	1.68	2.34	2.17	3.4	1.94	1.89	1.97	1.94	1.95	2.02	1.96 [1.94–2.13]
Heart rate [bpm]	57	70	55	59	70	63	72	68	75	84	67 ± 8.9
Ejection fraction [%]	61	33	30	26	35	22	38	24	49	18	34 ± 13.1
Stroke volume [ml]	91	76	55	52	70	34	69	53	104	45	65 ± 21.5
EDV [ml]	150	229	184	199	202	158	183	224	212	245	199 ± 20.5
ESV [ml]	59	153	130	147	132	124	114	171	108	201	134 ± 38.3
Mitral regurgitation (MR) fraction [%]	64	62	47	50	56	30	34	55	47	65	51 ± 12.0
E-wave flow [ml/s]	456	490	311	494	586	344	368	425	610	664	475 ± 118.0

For plausibility check, the simulation results are compared to clinical routine data of 21 patients (age: 79±6 years, BSA: 1.83[1.72–2.00], *n* = 9 female, MR grade II–IV) who received mitral TEER to treat MR of various causes (primary, secondary, and mixed). Mitral orifice areas were evaluated by planimetric measurements from 3D TEE images. Maximum and mean velocity and mitral pressure gradients were measured using continuous wave Doppler echocardiography images. All TEE images were acquired peri-operatively in routine practice with a GE Vivid E95 Ultrasound machine (GE Healthcare, Chicago, Illinois, USA).

### 2.2. Image processing

Pre-operative TEE images of the simulation cohort acquired with a GE Vivid E9 Ultrasound machine (GE Healthcare, Chicago, Illinois, USA) were processed using the software Tomtec Arena (Tomtec Imaging Systems GmbH, Unterschleissheim, Germany). Imaging was performed with a synchronized electrocardiogram. Volumetric TEE sequences that were used for segmentation had a time resolution of 23 ± 5.5 frames per cycle. The automated LV-Analysis tool of Tomtec Arena was used to segment the LV over an entire cycle from 4D TEE images. Manual corrections were applied at the end-diastolic phase to improve the accuracy of the automated segmentation. A segmentation of the mitral valve in the early diastolic phase was obtained by manual adaptation of the automated valve segmentation during systole by means of the 4D MV-Assessment tool (Tomtec Arena). The MV commissure definition results from the automated systolic segmentation of Tomtec and was not changed during manual adaption. Only the leaflet reconstruction of the initial segmentation was adapted to the open state in a frame during diastole by moving the segmentation spline until it overlapped the leaflets.

Both MV and LV segmentation were exported as triangulated surface meshes in the STL format. The initial segmentation of Tomtec has a low spatial resolution. To enhance surface quality, the MV geometries are remeshed and smoothed, after which they have an average edge length of 1.3–1.8 mm. Detailed mesh statistics can be found in the Supplementary material. The manual adaptation may be associated with uncertainties in the resulting valve geometries. We investigated the effect of inter-user variability on manual valve segmentation by experts and its influence on CFD-computed hemodynamic results in our previous work (31, 32). Results showed that the proportional variation in pressure drop and maximal velocities is smaller than the expected uncertainty of ultrasound measurements of these parameters (33).

### 2.3. Virtual TEER of the mitral valve

**Figure 2:**
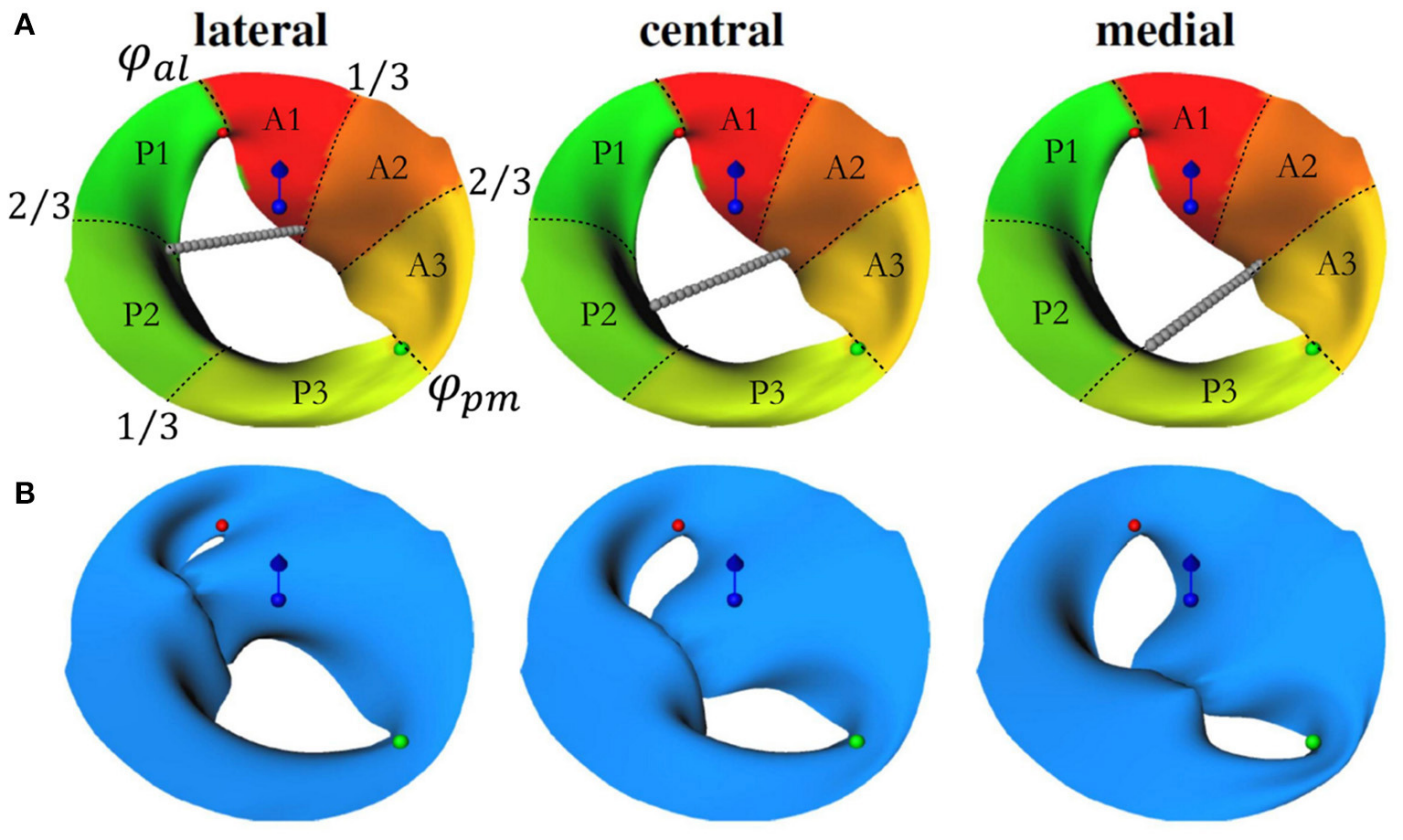
**(A)** Visualization of the automated setup for the device positioning. Red and green spheres depict the anterolateral (at φ_*al*_ = 0) and posteriomedial commissures (at φ_*pm*_). Sectors in red-yellow mark the anterior leaflet segments (φ∈[0, φ_*pm*_]), yellow-green sectors mark the posterior leaflet segments (φ∈[φ_*pm*_, 2π]). The center of gravity of the annulus is marked by the blue sphere while the blue vector shows the annulus plane normal. The gray chains of spheres indicate lateral, central, and medial device positions and depict which sectors are to be connected in the simulation. **(B)** Geometries after virtual TEER and slight postprocessing. Similar to **(A)**, commissure positions, annulus plane normal, and center of gravity are depicted.

To perform an automated virtual TEER at comparable positions, commissure points of the segmentation were added as landmarks to each valve data set. This information was used to divide the reconstructed valve surface into anterior and posterior segments and into sectors A1, A2, A3, P1, P2, and P3 (see Figure 2A). For this, the vertex positions of the valve mesh were transformed into a cylindrical coordinate system (*r*, φ, *z*) in which the height axis (*z*-component) was aligned with the normal vector of the annulus plane (blue vector in Figure 2). The origin of the new coordinate system was set to the center of gravity of the annulus (blue sphere in Figure 2). The zero angle component φ_*al*_ = 0 was aligned with the anterolateral commissure position (red sphere in Figure 2), and the angle component of the posteromedial commissure position φ_*pm*_ was determined (green sphere in Figure 2). Using this definition, the valve surface was segmented into anterior (all vertices with coordinates ∈{(*r*, φ, *z*) | φ∈[0, φ_*pm*_]}) and posterior leaflets (vertices ∈{(*r*, φ, *z*) | φ∈[φ_*pm*_, 2π]}) and into the six sectors {A, P}{1, 2, 3}. The boundaries between the sectors were located at $\frac{1}{3}$ and $\frac{2}{3}$ arc length of the respective leaflet segment (Figure 2A). Three device positions were defined to be at $\left\{\frac{1}{3},\frac{1}{2},\frac{2}{3}\right\}$ arc length of the respective leaflet segment (left to right in Figure 2). Oriented at measurements of a commercially available TEER device, we defined the diameter of the grasped leaflet area to be 5 mm. Using the three arc length positions and the diameter, device placing areas were defined at the free ends of the leaflets.

The virtual TEER process is simulated by means of our previously developed approach (34–36). The approach simulates mitral valve dynamics with position-based dynamics (PBD), which is an efficient simulation technique designed for real time computer graphics applications. It uses a mass-constraint system to model elastic deformations where mesh vertices are represented as point masses with position and velocity. The elastic material behavior of mitral valves can be approximated by multiple constraints modeling distance, bending, and area conservation constraints. Dirichlet boundary conditions, external forces, and collision constraints model the final dynamics, e.g., simulating the closing of the mitral valve during systole. To model virtual TEER, we needed to relate the opposite device placing areas to each other. For this, we used ray casting. Rays originating from one of the device placing areas cast in the direction of the other leaflet were used to define springs between opposite device placing areas. In PBD, springs are modeled as positional constraints between two mesh vertices. For virtual device closure, spring rest lengths were set to zero. Using the zero rest length modeling for the springs, the opposite leaflets were deformed toward and opposite device placing areas were mapped onto each other in the simulation (see Figure 2B). We applied the same material parameterization as previously published in Walczak et al. (36) for simulating the closing of healthy and pathological mitral valves. No fiber directions, non-linearities, calcifications, chordae tendineae, papillary muscles, or trabeculae were modeled. Collision constraints prevented self-intersections. No external forces were applied. The annulus contour was kept fixed.

### 2.4. CFD simulations

A total of 70 CFD simulations were performed in Simcenter STAR-CCM+ (Siemens Industries Digital Software. Simcenter STAR-CCM+, version 2020.1, Siemens 2020) using a quasi-stationary approach to mimic peak diastolic flow, according to the same principles as in our previous work (30, 32). Continuity equation for incompressible fluids (Equation 1) and momentum equations (Equation 2) were discretized by means of a finite volume formulation and an implicit second-order scheme with Δ*t* = 10^−4^ s was applied for temporal discretization. Blood was modeled as an incompressible non-Newtonian generalized Carreau-Yasuda fluid with dynamic viscosity (Equation 3) with η_∞_ = 0.0035Pa·s, η_0_ = 0.16 Pa·s, λ = 8.2 s, *n* = 0.2128, *a* = 0.64 [see (37)].


(1) $$\begin{array}{r} \nabla \cdot \vec{v}=0 \end{array}$$

(2) $$\begin{array}{r} \rho \left(\frac{\partial \vec{v}}{\partial t}+\left(\vec{v}\cdot \nabla \right)\vec{v}\right)=-\nabla p+\eta \Delta \vec{v}+\vec{f} \end{array}$$

(3) $$\begin{array}{r} \eta \left(\overset{∙}{\gamma }\right)=\eta_{\infty }+\left(\eta_{0}-\eta_{\infty }\right){\left(1+{\left(\lambda \overset{∙}{\gamma }\right)}^{a}\right)}^{\frac{n-1}{a}} \end{array}$$

Mesh base size was set to 1.0 mm with a refinement in the MVA down to 0.25 mm to ensure sufficient spatial resolution of expected detachment phenomena and acceleration at the leaflet tips. The conducted mesh independence study can be found in the Supplementary material. The atrium was modeled as a funnel with a single inlet which was set to a zero pressure boundary condition while the funnel wall, as well as the mitral valve, were assigned with a no-slip boundary condition and kept fixed. According to the referenced wall model (30), the ventricle serves as a velocity outlet representing the instantaneous LV movement at peak diastole.

### 2.5. Patient-specific boundary condition and modeling of hemodynamic parameters

**Figure 3:**
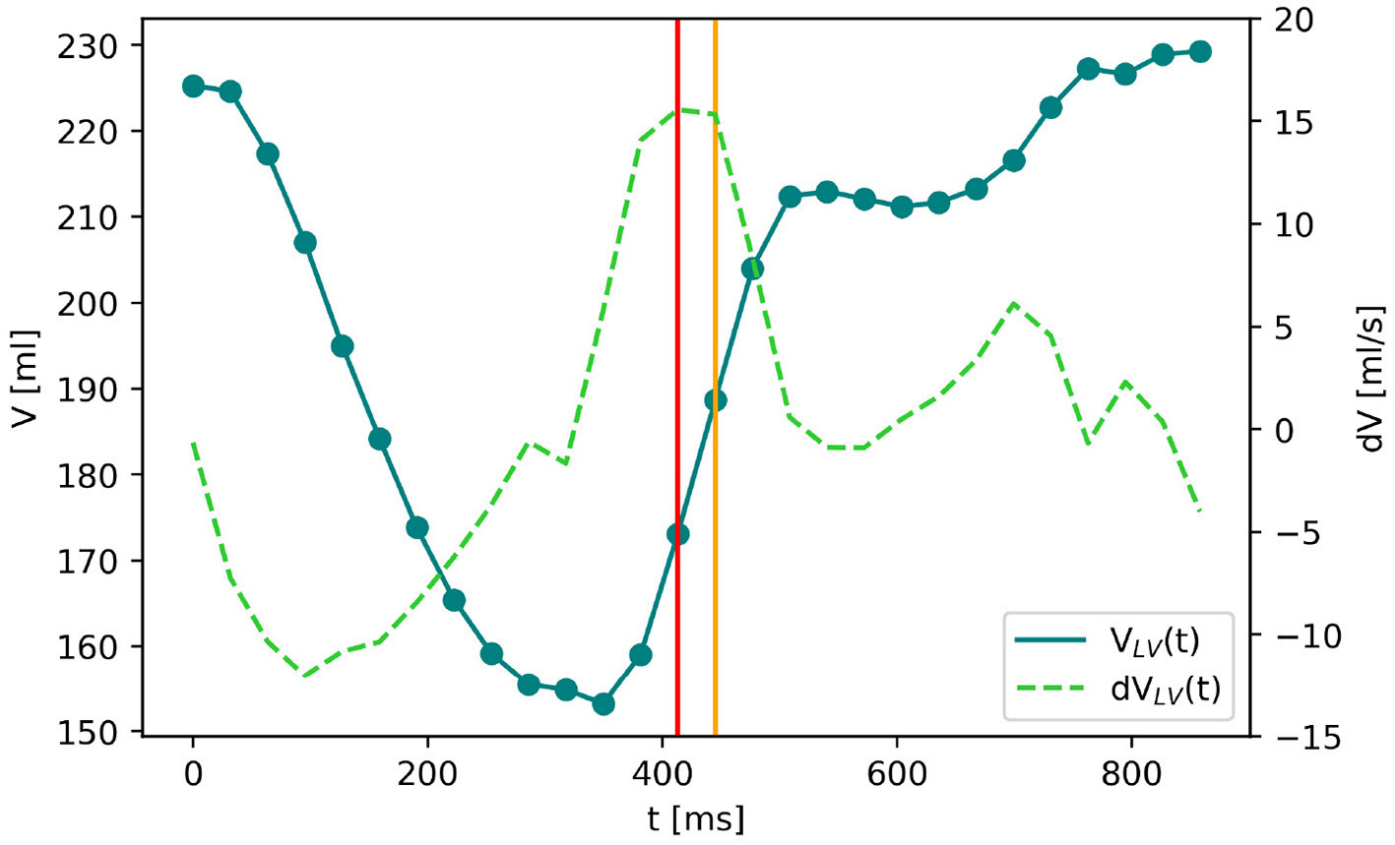
The blue solid line shows the volume curve *V*_*LV*_(*t*) of the left ventricle of Patient 2 over the time of one heartbeat. The dots are the time frames of echocardiographic imaging. *dV*_*LV*_(*t*) (green dashed line) denotes the LV volume change between two subsequent time frames. The biggest positive volume change (marked by the red vertical line) indicates the E-wave which is the moment of maximal blood flow through the mitral valve during diastole. The LV segmentations at this and the following time point (yellow vertical line) are chosen to create the surface distance map for the boundary condition of the computational fluid dynamics (CFD) simulation.

**Figure 4:**
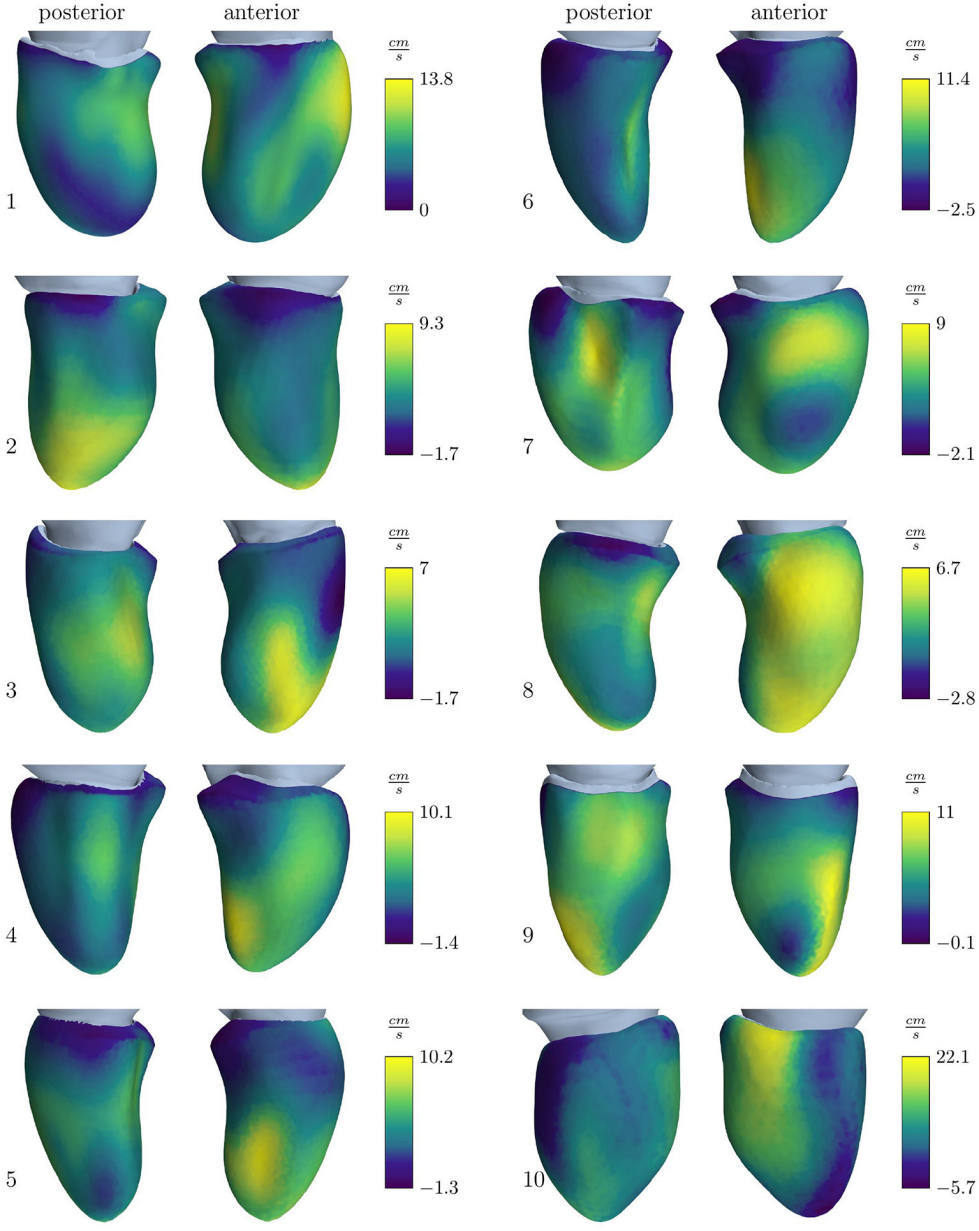
Velocity outlet boundary condition for the simulation cohort resulting to obtain the E-wave flow measured by volumetric analysis. Positive values stand for an outward directed flow representing wall movement away from the center, while negative values represent a movement toward the center.

For the quasi-stationary simulations at the peak-E wave, a boundary condition is required to represent the instantaneous wall movement and therewith induced volume change of the LV, which results in the peak E-wave flow rate (474.9 ± 117.9 ml/s, listed for each case in Table 1). The above mentioned automated LV analysis performed in Tomtec Arena was used to obtain the volume curve as exemplarily shown for Patient 2 in Figure 3. These image data were also taken to receive end-diastolic and end-systolic LV volume, resulting in stroke volume and ejection fraction as listed in Table 1. The peak diastolic time point was identified by the largest positive volume difference Δ*V* between two consecutive segmentations Δ*t* (note the two vertical lines in Figure 3). Segmented LV geometries of these two time points were used to derive a patient specific boundary condition for the quasi-stationary CFD simulations by mapping the flow rate at peak E-wave by means of a distance map of the LV geometries at the respective time points. Therefore, the LV segmentations were exported as triangulated meshes in the STL format, and both geometries were aligned with the annulus center in the global origin by means of the software Blender (38). This step simplifies further alignment of the mitral valve and definition of internal coordinate systems in the CFD simulation setup. A surface distance map was calculated containing the distances in the normal direction from the LV with a smaller volume toward the other. In the CFD simulations, a velocity outlet boundary condition was applied at the LV wall. A surface distance map between the two chosen time steps was used to weight the fluid velocity at the walls such that the resulting volume flow in the simulations matched the instantaneous blood flow at peak E-wave (compare Figure 4).

The peak E-wave flow rate of the clinical comparison data was estimated by multiplying the echo Doppler measured maximal velocity with the MVA, measured by planimetry: ${\overset{∙}{V}}_{E-wave}=v_{max}\cdot MVA$. This delivered similar results as for the simulation cohort (447.6 ± 107.6 ml/s). After TEER, the clinical data had a residual MR of trace to mild and showed in average a drop of peak E-wave flow rate by 23%, resulting in 372.9 ± 115.9 ml/s. However, no significant linear regression was found between pre- and post-interventional flow rate values. Since the post-interventional simulations after virtual TEER are based on pre-interventional data only, we assume the same average drop in flow rates as observable in the clinical data. Hence, the post-interventional flow rates of the simulation cohort under the conditions of rest are 77% of the pre-interventional flow rates. With the observation, that the heart was capable of affording a higher flow rate pre-interventionally, we further presume the pre-interventional flow rate to serve as a reasonable estimation to mimic conditions of moderate stress after virtual TEER.

**Figure 5:**
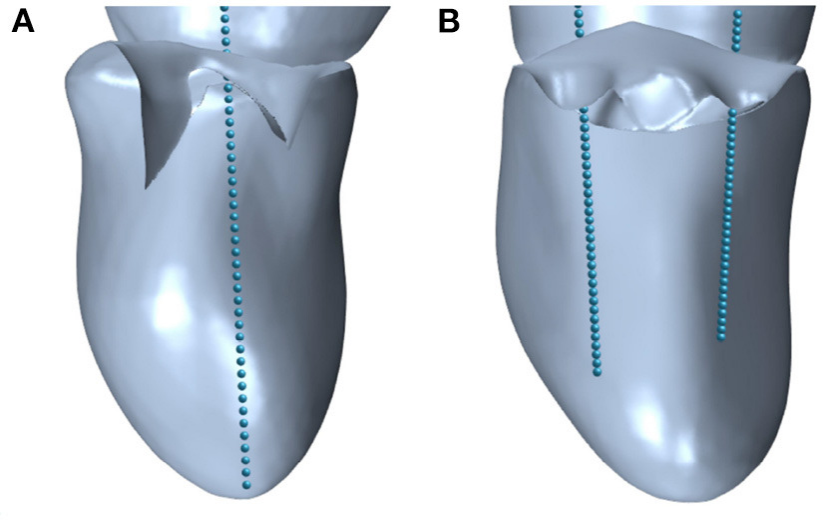
Line probes defined in STAR-CCM+ to extract the pressure in **(A)** pre-interventional geometries with single orifice and **(B)** post-interventional geometries with double orifice.

During TEER interventions, three of the basic hemodynamic surveillance parameters are the MVA, mean and maximum velocities, which are measured by ultrasound, and mean and maximum MPG, which are estimated by means of the simplified Bernoulli Equation (Equation 4). The MVA is an important indicator for assessing the suitability of a TEER procedure for a patient since guidelines recommend to only consider patients with a MVA>4 cm^2^ for TEER (14). During and after the procedure, MVA and MPG are used to judge the risk of MS. These parameters were also analyzed in the simulation cases of this study. Thereby, the effective MVA before and after virtual TEER was measured by means of the shrink-wrap approach developed by Razafindrazaka et al. (39). Maximum velocities were monitored during the simulation time. They occured at the shear layer of the leaflet tips during early simulation time and travelled with the vortex structures toward the mid ventricle. The static pressure was numerically calculated by solving the Poisson-Equation within STAR-CCM+ since the simulations work with an incompressible fluid. It was extracted along a line probe that is set between the annulus center and the apex for all cases before virtual TEER as shown in Figure 5A. After virtual TEER, a line probe was placed in each opening of the double orifice (Figure 5B) and the results of both probes were averaged. Note that in clinical routine mean and maximum of both velocity and MPG are available. The simulations performed with the presented setup, however, only allow for the assessment of maximum values of velocities and pressure gradients.


(4) $$\begin{array}{r} \Delta P\left[mmHg\right]=4\left[\frac{mmHg\cdot s^{2}}{m^{2}}\right]\cdot v_{max}^{2}\left[\frac{m^{2}}{s^{2}}\right] \end{array}$$

### 2.6. Statistical analysis

Statistical analysis of simulated and clinical data was performed using IBM SPSS Statistics 28 software (IBM Company, USA). Mean and SD were reported for normally distributed parameters as assessed by the Shapiro-Wilk test due to the high statistical power of the test and due to the small size of both cohorts. Otherwise, median and interquartile [IQR] ranges were used. The two-tailed student's *t*-test was used to test for significant differences within normally distributed parameter differences, while the Mann–Whitney U and Wilcoxon signed-rank tests were used for testing non-normally distributed parameter differences. Paired tests were used to compare differences between pre- and post-treatment and between different device positions. All tests used a standard significance level of 0.05.

## 3. Results

In the following, results of the simulations before and after virtual TEER under conditions of rest and stress are presented and compared to clinical routine data. First, the influence of virtual TEER on the MVA was analyzed. Furthermore, the results of image data analysis delivering the boundary conditions for CFD and hemodynamic results of the CFD simulations themselves were investigated. Table 2 summarizes the results.

**Table 2:** Hemodynamic results after virtual trans-catheter edge-to-edge repair (TEER) at rest and stress conditions for different device positions.

		**Pre**	**Post_*all*_**	**Lateral**	**Central**	**Medial**
MVA [cm^2^]	Simulation	7.50 ± 1.62	2.56 ± 0.63	2.65 ± 0.68	2.30 ± 0.58	2.72 ± 0.62
	Clinical routine	4.75 ± 1.00	2.81 ± 0.80	–	–	–
v_*max*_ [m/s]	Simulation rest	0.90 ± 0.16	2.04 ± 0.45	1.94 ± 0.60	2.20 ± 0.38	1.97 ± 0.32
	Simulation stress	–	2.59 ± 0.57	2.41 ± 0.57	2.78 ± 0.48	2.59 ± 0.63
	Clinical routine	0.96 ± 0.21	1.33 ± 0.21	–	–	–
MPG [mmHg]	Simulation rest	1.46 ± 0.63	16.30 ± 9.0	14.92 ± 11.65	19.51 ± 8.54	14.47 ± 5.90
	Simulation stress	–	27.36 ± 13.34	23.01 ± 13.48	32.14 ± 12.31	26.94 ± 14.23
	Clinical routine	3.85 ± 1.72	7.24 ± 2.28	–	–	–

### 3.1. Mitral valve area

Placing a TEER device, virtually or in a real procedure, has the two effects of both reducing the mitral orifice area and usually converting the single orifice of the native valve into a double orifice. The projected MVAs of the segmented mitral valve geometries measure a mean and SD of 7.50 ± 1.62 cm^2^ and reduce to 2.56 ± 0.63 cm^2^ after virtual TEER when averaging over all device positions. Comparison of the remaining MVA after virtual device placement at different positions shows that central positions lead to a significantly stronger reduction of MVA than eccentric positioning of the device (paired *t*-test) at either lateral (*p* = 0.002) or medial (*p* = 0.035) segments of the leaflets. A virtual TEER at the A1-P1 segments (lateral) leads to a mean MVA of 2.65 ± 0.68 cm^2^ and A3-P3 (medial) leads to a mean MVA of 2.72 ± 0.62 cm^2^. This is a reduction of MVA to 35 and 36% of the original MVA, respectively. Central device positioning at segments A2-P2 reduces the MVA to 31%, measuring 2.30 ± 0.58 cm^2^. This leads to an average difference of 0.35–0.42 cm^2^ between central and eccentric device positions (see Figure 7A). Having a closer look at the MVAs of the single patient cases after virtual TEER, two of them get close to the clinical definition of severe stenosis (MVA <1.5 cm^2^) according to (3). Patient 3, e.g., shows a pre-interventional MVA of 5.58 cm^2^ which reduces to 1.54 cm^2^ after central device placement while lateral and medial positions lead to 2.02 and 1.79 cm^2^, respectively. With a pre-interventional MVA of 5.92 cm^2^, Patient 6 exhibits remaining MVAs of 1.67 and 1.69 cm^2^ after central and medial device position, while lateral positioning entails a much bigger MVA of 2.43 cm^2^. All other cases maintain MVAs of >1.7 cm^2^, independent of the device position, and would therefore not be judged as severe stenosis cases according to the guidelines (3). A brief comparison to clinical routine data shows good agreement regarding measurements of post-interventional MVA, which are 2.53 ± 0.79 cm^2^. Pre-interventional MVA, on the contrary, measures significantly smaller values of 4.75 ± 0.99 cm^2^.

**Figure 6:**
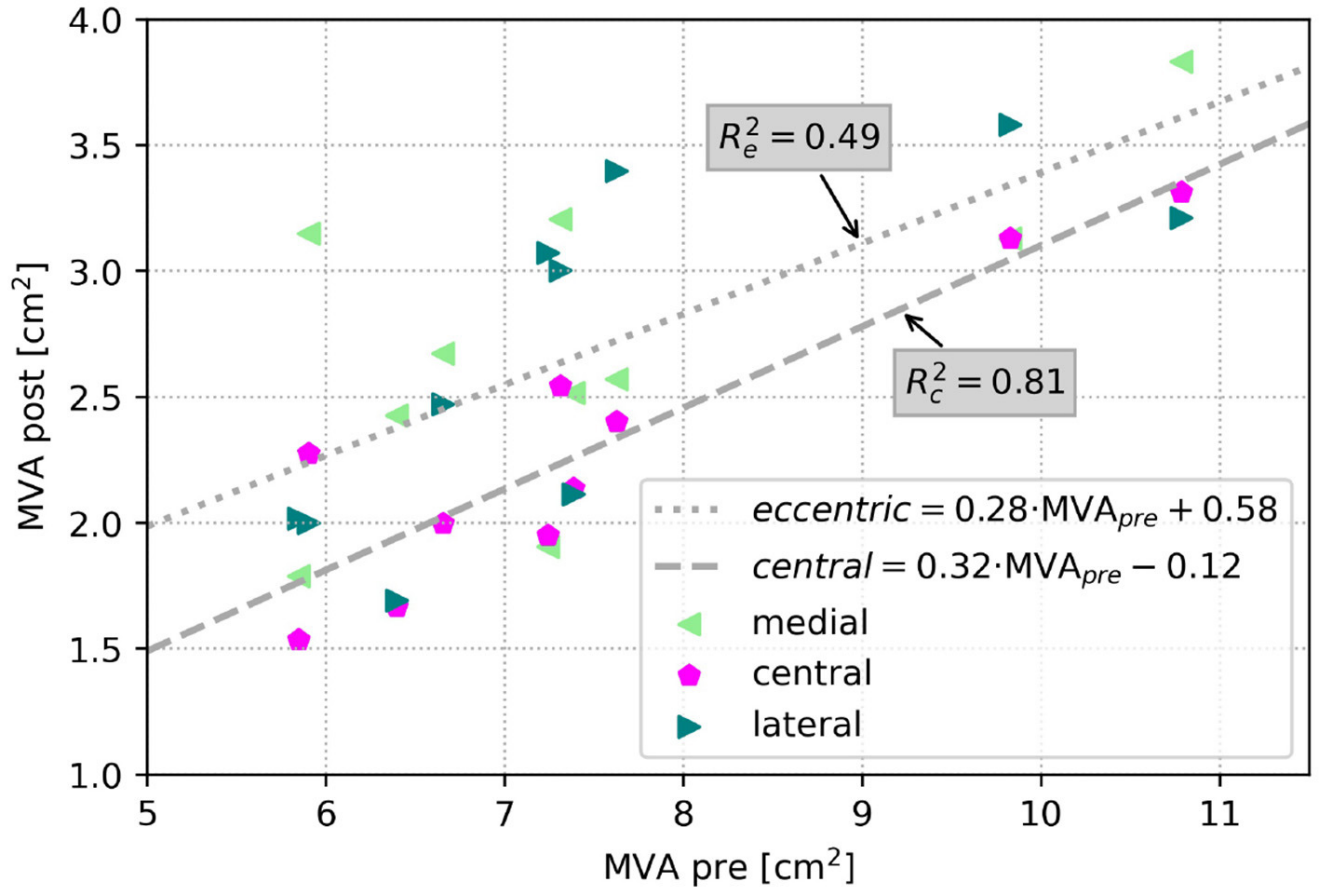
Scatter plot of MVA in the simulation cohort after virtual TEER at all positions respective to MVA before virtual treatment. The dashed line shows a regression of central device positions (R${}_{c}^{2}$ = 0.81), and the dotted line shows the regression for eccentric device positions (R${}_{e}^{2}$ = 0.49).

Figure 6 depicts respective changes of MVAs in the simulation cohort for each patient after virtual TEER at central or eccentric (lateral and medial) positions in a scatter plot. Regressions for central and eccentric positions show a low coefficient of determination (*R*${}_{e}^{2}$ = 0.49), hence large scattering for eccentric device positions. In contrast, MVA after central virtual TEER shows lower scattering with *R*${}_{c}^{2}$ = 0.81. For an illustration of the mitral valve geometries and detailed MVA results of all cases at all device positions the reader is referred to the Supplementary material.

### 3.2. Hemodynamic parameters

The quantitative analysis of hemodynamic parameters is focused on the simulations results of maximal velocities and pressure gradients at peak E-wave flow. Further, the intraventricular flow structures evolving at different device placement locations are looked at qualitatively for exemplary cases.

#### 3.2.1. Maximum velocity and pressure gradient

Prior to virtual TEER, the maximal velocities in the simulation cohort were 0.90 ± 0.16 m/s. The simulations after virtual TEER at all positions showed that maximal velocities rise to 2.04 ± 0.45 m/s at rest conditions and significantly higher to 2.59 ± 0.57 m/s under conditions of light stress. This is an increase of 127% at rest and 188% under stress in relation to the pre-interventional simulations. In the clinical data, on the contrary, the maximal velocities only rose by 38% from 0.96 ± 0.21 m/s before to 1.33 ± 0.21 m/s after the TEER therapy.

**Figure 7:**
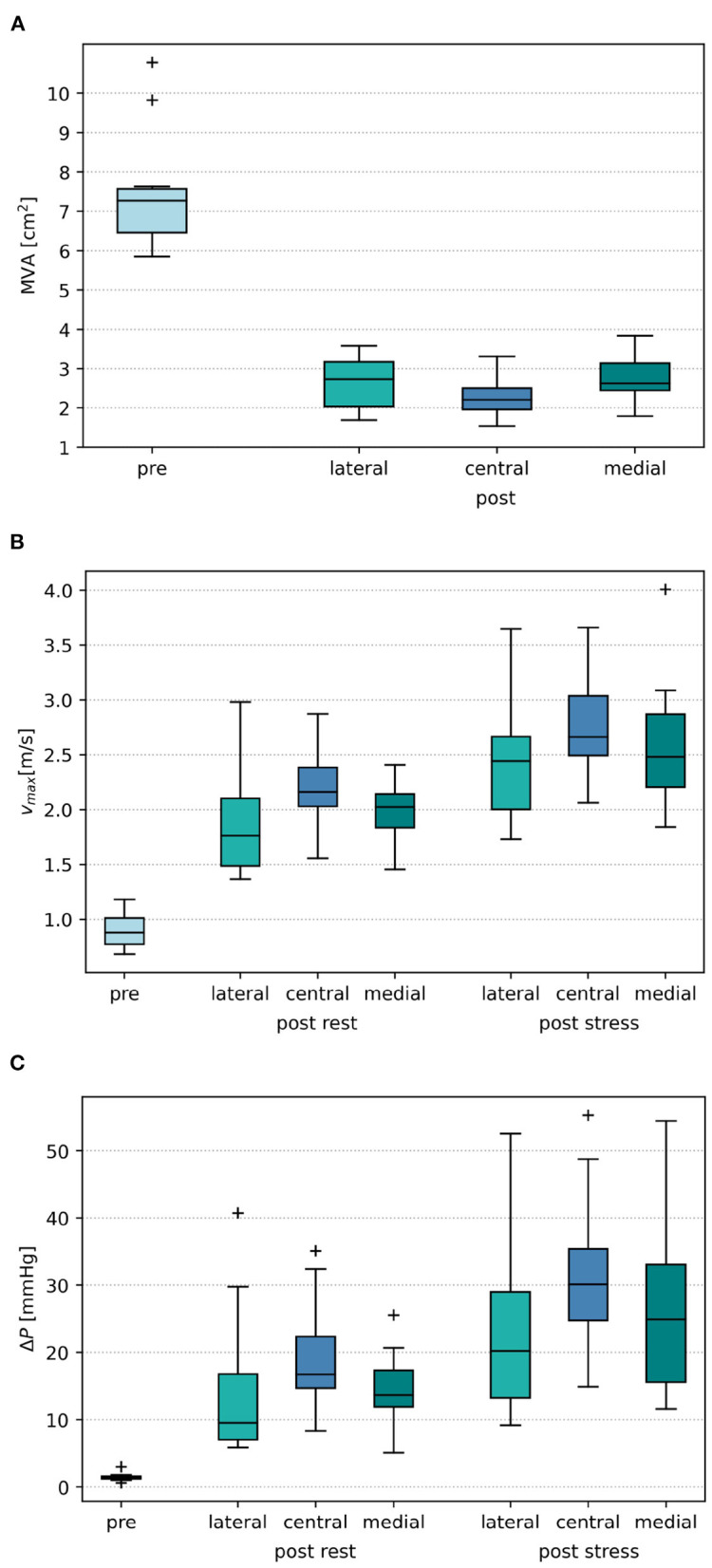
**(A)** Boxplot of mitral orifice area (MVA) before virtual treatment (pre) and after virtual TEER placement at the lateral, central, and medial position, respectively. **(B)** Boxplot of pre- and post-interventional maximal velocities *v*_*max*_. **(C)** Boxplot of pre- and post-interventional mitral pressure gradient Δ*P*.

Figure 7B shows that central device positions lead to a significantly higher rise in maximal velocities than eccentric device positions. At rest, maximal velocities show a larger increase of 0.23–0.27 m/s after central virtual TEER, than after eccentric device placement at the lateral or medial position. Stress boundary conditions in combination with a central device position, averagely resulted in 0.19–0.37 m/s higher maximal velocities than stress simulations with eccentric device positions. These results are listed in detail in Table 2.

**Figure 8:**
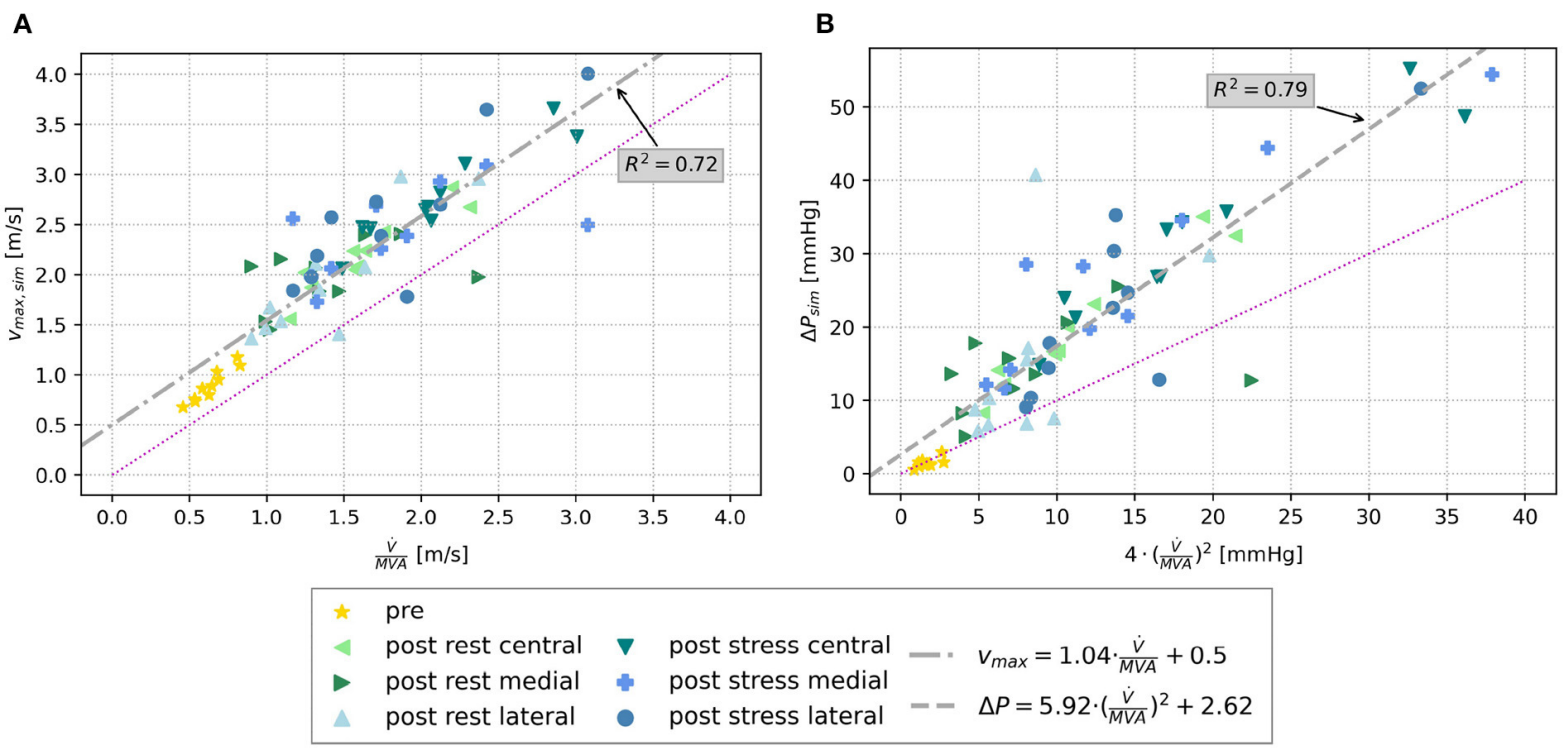
**(A)** Scatter plot of simulated maximal velocities pre and post virtual TEER at rest and stress, respectively, over the estimated maximal velocity using volume flow and MVA. **(B)** Scatter plot of simulated MPG (Δ*P*) over the estimated MPG by means of the simplified Bernoulli-Equation (Equation 4). The bisector is indicated by the magenta line in both plots, and a regression line is drawn in gray.

Similar tendencies are seen for the MPG. It increased from 1.46 ± 0.63 mmHg before virtual TEER to 16.30 ± 9.00 mmHg at rest and 27.36 ± 13.34 under stress considering all device positions. Pre-interventional MPGs of the clinical comparison data were significantly higher with 3.85 ± 1.72 mmHg and lower after the procedure with 7.24 ± 2.28 mmHg. Figure 7C shows a stronger increase of MPG at central compared to eccentric device position. In detail, the MPG rose on average roughly 5 mmHg higher after central device positioning than after eccentric virtual TEER at rest and about 5–9 mmHg higher under stress (Table 2). The stronger spread in measured velocities and pressure differences under conditions of stress is visible in Figures 7B,C. Regarding the objective of providing tools to support clinical therapy planning and decision making by predicting possible patient outcomes after treatment, it is important to consider habits of estimating hemodynamic parameters in clinical routine when comparing results of clinical and simulation outcomes. Therefore, simulated results are plotted over estimations, that can be drawn from easily accessible parameters, such as volume flow ($\overset{∙}{V}$) and MVA (Figure 8). The simulated values of velocity were, apart from few outliers, above the estimated values and regression of the point cloud is almost parallel to the angle bisector but shifted upward by 0.5 cm/s with R^2^ = 0.72 (Figure 8A). Estimations of the MPG and simulated values corresponded well for the pre-interventional cases, which are directly on the angle bisector of the plot (Figure 8B). Post-interventional cases, on the contrary, revealed considerably higher MPGs in simulations than estimated with the Bernoulli-Equation based on volume flow and MVAs.

#### 3.2.2. Ventricular flow structures

**Figure 9:**
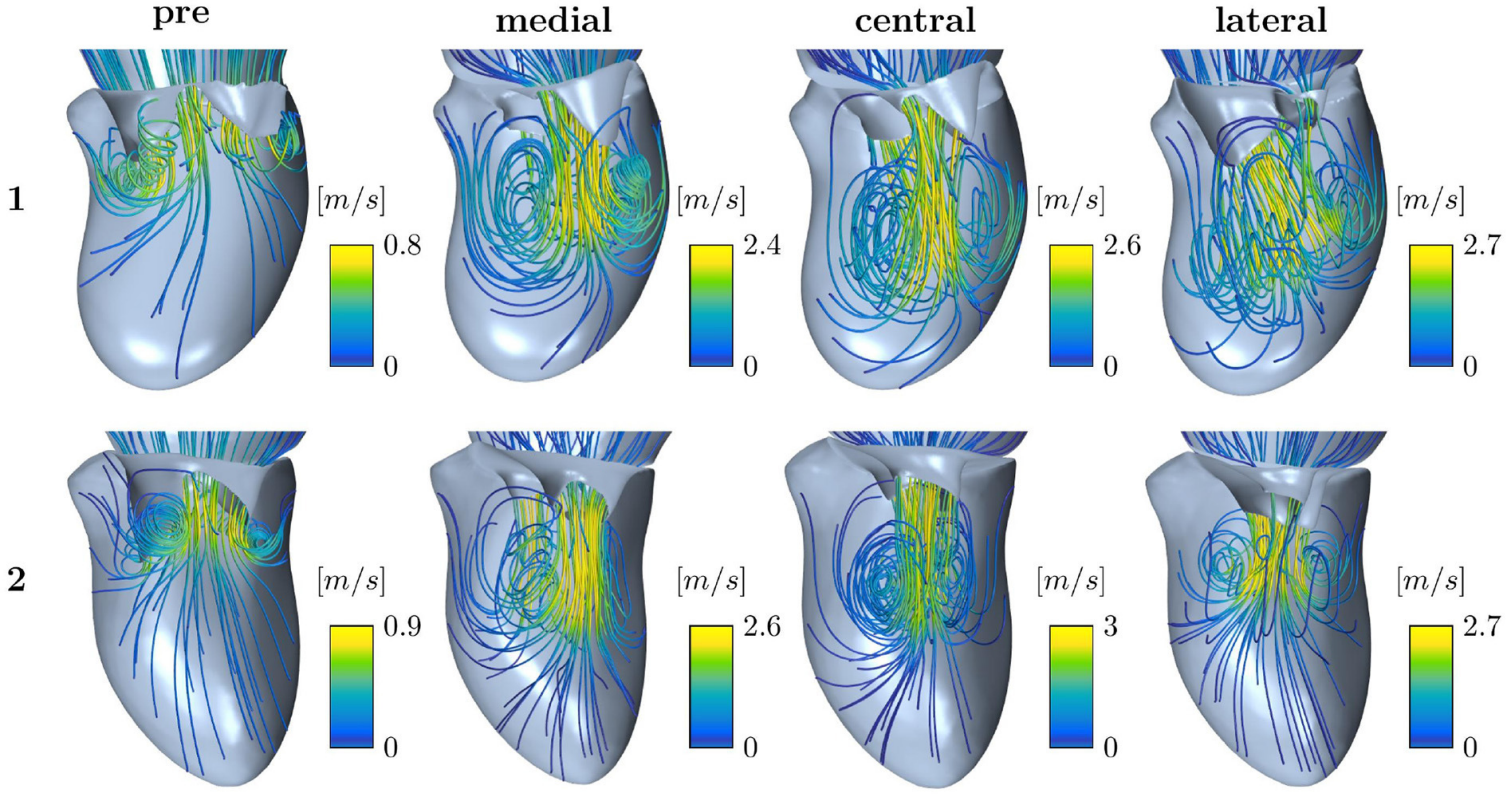
Streamline representation of the flow field for pre-interventional conditions and after virtual TEER at medial, central, and lateral device positions, respectively, for Patients 1 and 2.

Intraventricular flow structures can, in contrast to velocity and pressure gradients, only be compared qualitatively among different simulation setups since ultrasound data do not capture them. Figure 9 shows streamlines of the early diastolic inflow jet of Patients 1 and 2. Pre-interventional flow (left) is opposed to post-interventional flow after medial, central, and lateral device positions, respectively. At the leaflet tips, the jet is rolling up at the shear layer of the tips, developing the diastolic vortex ring. The velocity maxima are located in the center of the jet. All device positions cause a division of the pre-interventional single orifice area into a double orifice. Under identical boundary conditions, the device positions have a strong influence on the vortex formation and jet direction. For example in Patient 1, a medial device position leads to a stronger vortex than central or lateral device positions, whereas lateral positioning directs the diastolic jet more towards the inerolateral LV wall. In this case, the maximum velocities differ only weakly between the device positions. For Patient 2, however, the effect of stronger acceleration after central compared to eccentric device position is more clearly exhibited than the effect on the jet direction. Nevertheless, after lateral device placement, the jet at the anterior LV wall turns out stronger, while a medial device positioning enhances the jet at the inferior LV wall. Illustrations of the flow structures of all patients can be found in the Supplementary material. Considering all patient cases, it becomes clear, that the device position has various effects, not only on changes in velocity and MPG but also on redirecting the diastolic jet and acting on the vortex formation and development.

## 4. Discussion

In this study, we established a workflow of virtual TEER device placement in patient-specific mitral valve geometries and modeled the impact of the device position on diastolic hemodynamics under rest and stress conditions. Our key findings are that MVA reduces most at central device positions, which leads to a stronger increase in hemodynamic parameters associated with iatrogenic mitral stenosis. Vortex structures are highly dependent on individual valve morphology and boundary conditions. Results and clinical application of the workflow are discussed in the following.

### 4.1. Mitral valve area

Virtual TEER treatment simulations allow an estimation of post-treatment MVA. We found that central device positions lead to a stronger reduction of MVA (Figure 7A). On average, central device positions led to a 0.36–0.42 cm^2^ smaller residual MVA after virtual TEER than eccentric device positions. This diverges from the results of Kassar et al. (20), who found the biggest reduction of MVA in a slightly eccentric position. Focusing on individual cases of the cohort, two out of ten can be considered borderline cases. They reach or come close to the cut-off range for severe mitral stenosis of MVA <1.5 cm^2^ (3) after central virtual device placement, whereas eccentric device positions result in MVAs above that limit. A virtual treatment tool as presented here could both help to identify such borderline cases and support the careful planning of device positioning with regard to residual MVA.

Furthermore, we were able to show that residual MVA in the simulated cases is also better predictable after central than after eccentric virtual device placement by exhibiting a stronger linear correlation with the pre-interventional MVA (compare Figure 6). This leads to the assumption that residual MVA after eccentric device positioning is more dependent on individual valve morphology, such as annulus area, leaflet length, and coaptation area.

While post-interventional MVAs of the simulated cases align well with the clinically measured MVA after TEER, the pre-interventional MVAs are significantly bigger in the simulation data, even though the planimetric measurement is suspected to overestimate the MVA (20). This may be caused by an overestimation of MVA by the shrink-wrap algorithm (39), which is stronger for single than for double orifices. Systematic studies on the difference in MVA measurements from ultrasound and segmented valve geometries are therefore vital.

### 4.2. Hemodynamics after virtual TEER

Analysis of pre- and post-treatment hemodynamics found that maximal velocities and MPG increased more after central than after eccentric virtual device placement (see Figure 7). This corresponds to the findings of smaller MVA for central device positions. However, a wide spread in the data is observed suggesting a strong dependence on individual factors. With regard to clinical applicability, we tested whether the maximal velocity and MPG could be predicted by the individual volume flow and MVA only and observed good correlations (see Figure 8). The ratio of peak E-wave volume flow and MVA is a simple estimation for the average velocity necessary to obtain a certain flow rate through an orifice area. In the diastolic flow, maximal velocities appear first at the rolled up shear layer close to the leaflet tips. After formation and progression of the diastolic jet, maximal velocities are found in the center of the jet. This also explains the discrepancy between pre- and post-interventional MPG. Since the pre-interventional vortex ring and jet are not fully developed yet, pressure measurements taken at the centerline do not capture maximal velocities at the leaflet tips (Figure 9). On the contrary, clinical pressure gradients are estimated by using the Bernoulli equation.

Intraventricular flow structures are discussed to act on cardiac efficiency (40) and CFD provides a quantitative tool for further investigations toward this question in cohorts of MR patients receiving mitral TEER treatment. Our results show that flow patterns vary widely between the simulated cases and are not only influenced by the device position but also by the valve morphology and the boundary conditions including LV shape and LV contraction patterns (Figure 4).

It can be summarized, that simple considerations allow estimating the resulting maximal velocities and MPG from MVA and peak-E wave flow rate. CFD may therefore not be necessary. However, when investigating the impact of a TEER device on the flow structures, which have been shown to be very individual, CFD is a suitable tool to use. The need for a more complex CFD modeling, assessing the whole heart cycle or the whole diastolic phase instead of modeling the peak E-wave should be evaluated in future studies.

### 4.3. Clinical application

Therapy planning tools for TEER interventions are tremendously needed when it comes to device positioning, choice of device, and outcome prediction on an individual patient level. Ideally, such tools do not only help in procedure planning but also identify borderline cases and assess the risk of iatrogenic MS. This may be approached on the level of MVA estimation regarding geometric changes after TEER only or in combination with the consideration of hemodynamic parameters. To prevent an underestimation of MS, e.g., in patients with low-flow/low-gradient characteristics (21), outcome scenarios could be simulated by taking into account the following factors:

- Ventricular recovery, a potential improvement of left ventricular function, and a corresponding increase in diastolic blood flow.
- A reduction or absence or regurgitating volume, resulting in a decrease of the absolute stroke volume and counteracting rises in MPG.

Furthermore, intra-procedural stress testing might be replaced by simulation approaches, thus preventing the exposure of patients to additional risks.

Our workflow is based on data that can easily be acquired in clinical routine. Tomtec Arena used for valve segmentation is broadly available and integrated into clinical systems. The virtual TEER treatment with PBD is fast and easy to use. Further steps of the analysis could be automatized for better usability. CFD is considered to be a valuable method for development of prediction tools and to investigate academic questions rather than being used in clinical routine.

When considering the translation of simulation results into clinically interpretable data, it must be taken into account that ultrasound-measured velocity data is likely to underestimate velocities and pressure gradients compared to catheter-based measurements (41). A similar phenomenon is to be expected whenever checking clinical data against our simulation results. Since velocity acquisition with ultrasound is physically only possible for the component along the beam direction, an underestimation will increase with a rising inclination of the jet direction respective to the ultrasound beam as observable in case of double orifices after TEER treatment. It is further to mention that our simulations only allow for representation of maximum values of velocity and MPG, whereas clinical judgment is mostly based on mean values of those quantities. Riegel et al. (42) state however, that both mean and peak values of velocity and MPG are suitable for judging relevant iatrogenic MS after MR treatment.

Finally, it is important to point out that a general statement regarding the suitability of a certain device position is not possible. It highly depends on the etiology, the individual morphology of the MV, and potential adaptation to the treatment. The necessity of a patient-individual focus in systematic treatment planning tools is thereby underlined. Future work could enhance the understanding of relations between the number and position of TEER devices in combination with specific MR etiologies.

### 4.4. Limitations

Although there are not many simulation studies regarding TEER treatment with cohorts containing more than 10 cases, we still acknowledge the limitation of power and its related effects on statistical analysis. Furthermore, we have only examined deployment of one virtual TEER device, while many patients receive more than one device for the treatment of MR (43). We have refrained from simulating specific device products as a part of our simplified methodology. The quasi-stationary modeling approach means that all meshes are fixed. Detailed structures of the LV and MV apparatus, such as chordae, papillary muscles, and trabeculae, are neglected. Note that LV torsion has, according to the work of Vasudevan et al. (44) and Canè et al. (45), no crucial influence on the investigated parameters and has not been included in the model. Manual interaction in the segmentation process can moreover lead to user-dependent uncertainties which may influence the CFD results. Results of our previous investigation (32) show, however, that the proportional variation in pressure drop and maximal velocities is smaller than the expected uncertainty of ultrasound measurements of these parameters (33). Furthermore, the estimation of rest and stress boundary conditions have to be understood as estimations rather than validated relations. Finally, a direct validation with clinical data of the simulated cohort was not possible since the included patients did not receive the simulated treatment. Our comparison with the clinical cohort could be considered a plausibility check or feasibility proof.

## 5. Conclusion

High inter-individual and device location-dependent variability between morphometric and hemodynamic parameters before and after virtual TEER treatment was found in our study. Virtual TEER treatment using a position based dynamics approach combined with CFD seems to be a promising tool for predicting residual MVA and hemodynamic outcomes for varying device positions. Post-interventional scenarios can be simulated for varying flow conditions, associated with rest or stress. Once these flow conditions are validated, this enables stress testing without any additional risks for patients. The method could hence be used in the future to support treatment decision making and procedure planning. However, in order to translate the proposed approach into the clinical workflow, a clinical validation study is vital.

## Data availability statement

The data sets (geometries and applied boundary conditions) presented in this study are provided as open data on figshare: https://doi.org/10.6084/m9.figshare.19535047.v1.

## Ethics statement

The studies involving human participants were reviewed and approved by Ethics Committee of Charité—Universitätsmedizin Berlin. The patients/participants provided their written informed consent to participate in this study.

## Author contributions

KV and LG: conceptualization. KV and FB: data curation. KV, LW, and LG: formal analysis, methodology, and writing original draft. LG, UL, TK, and AH: funding acquisition and supervision. KV and LW: visualization. KV, FB, MR, and MK: investigation. All authors: review and editing, contributed to the article, and approved the submitted version.

## Funding

This work was part of the projects EurValve (funded by the European Union's Horizon 2020 research program, grant no. 689617), DSSMitral, and MINIMAKI, each funded by the German Federal Ministry of Education and Research (Grant nos. 03VP00851 and 16SV8649).

## Conflict of interest

Author FB received grant support from Abbott Laboratories (Chicago, USA). The remaining authors declare that the research was conducted in the absence of any commercial or financial relationships that could be construed as a potential conflict of interest.

## Publisher's note

All claims expressed in this article are solely those of the authors and do not necessarily represent those of their affiliated organizations, or those of the publisher, the editors and the reviewers. Any product that may be evaluated in this article, or claim that may be made by its manufacturer, is not guaranteed or endorsed by the publisher.
