# Supplementary material for: Effect of transcatheter edge-to-edge repair device position on diastolic hemodynamic parameters: An echocardiography-based simulation study
Source: Front Cardiovasc Med. 2022 Aug 24;9:915074. doi: 10.3389/fcvm.2022.915074 (PMC9449143; doi:10.3389/fcvm.2022.915074)
Supplement: Supplementary file 1 [file Data_Sheet_1.PDF]

Supplementary Material

1 MESH INDEPENDENCE FOR CFD CALCULATIONS

A mesh independence study was performed exemplary on Case 2 with central TEER device position in order to identify the necessary spacial resolution of the CFD simulations by congruent minimal computational effort. Table S1 gives an overview over the mesh metrics and resulting CPU solver time. All Simulations were performed on a workstation (AMD Ryzen 7 1700X Eight-Core 3.40 GHz Processor) to mimic routine application in the clinical field. Considering the results shown in Figure S1, base size of 1.0 mm was chosen as best compromise of accuracy vs. computational effort. The simulations take less than three hours on a local machine using 6 cores and provide an accuracy of > 95 % compared to the finest base size of 0.8 mm.

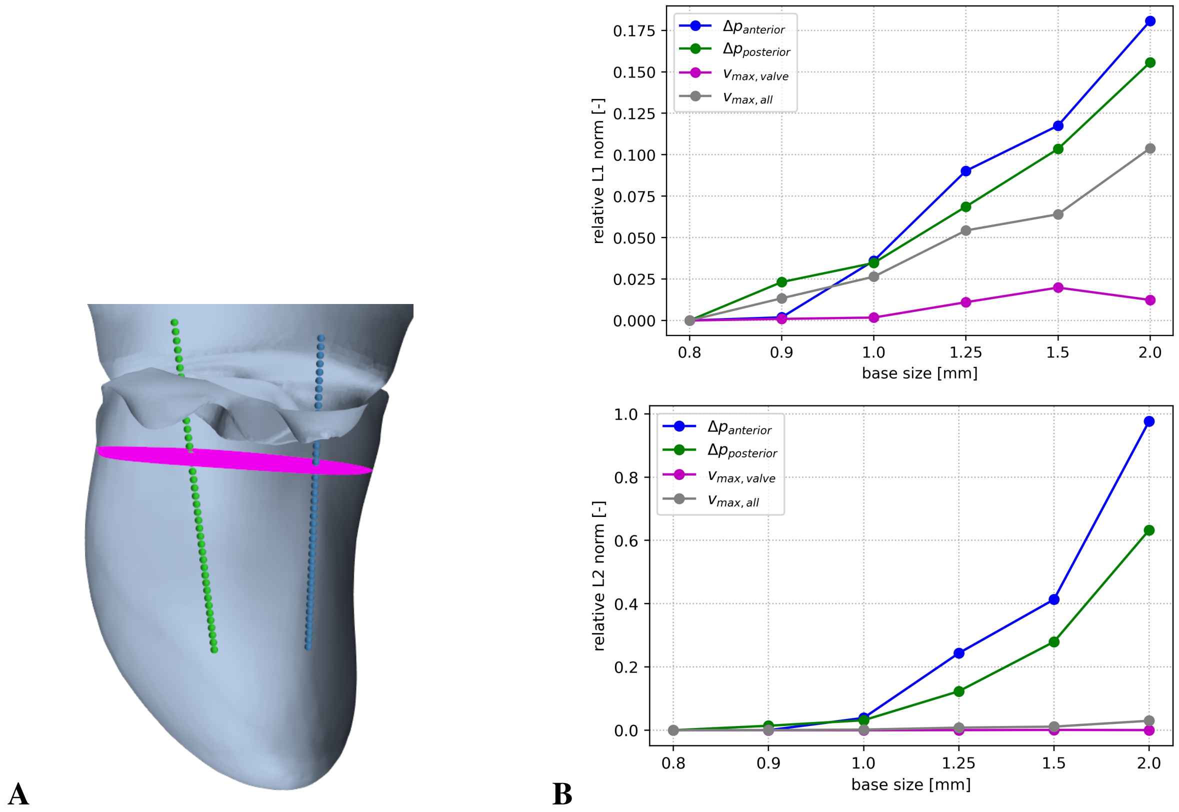

Figure S1: **(A)** Geometry of case 2 with TEER device in central position after virtual treatment. Maximal velocities were evaluated in the pink plane. Mitral pressure gradient (MPG) was measured along the line probes in the anterior (blue) and posterior (green) orifice. **(B)** Colors of the graphs refer to the colors in **A**. L1 and L2 errors of MPG and maximal velocities relative to the smallest base size of 0.8 mm are plotted over the compared base sizes.

2 SUPPLEMENTARY TABLES AND FIGURES

Statistical information of the mitral valve meshes used for the virtual TEER approach is listed in Table S2. The individual mitral valve area for each orifice and the leaflet area are listed in Table S3. Figure S2 shows the mitral valve geometry of all cases previous to the virtual TEER intervention and after placing

Table S1. This table lists the count of cells, faces and vertices for the respective base size with mesh refinement of 25 % in the mitral valve region. The needed CPU hours refer to a run time of 400 time steps with a second-order time discretization, a time step of $t = 10e - 4$ s and 15 inner iterations.

Base size [mm]	Cells	Faces	Vertices	Total CPU time [h]
0.8	1211530	7357540	6028674	32.92
0.9	913355	5488848	4478937	24.28
1.0	710692	4230198	3440758	17.69
1.25	419026	2447877	1979389	9.94
1.5	282907	1626581	1309479	4.77
2.0	151762	849796	678046	2.50

Table S2. Statistics for the edge length of the MV meshes in mm for each case. Each MV mesh has 1600 vertices, 4640 edges, and 3040 triangles.

Case	mean	std-dev	min	med	max
1	0.489228	1.29952	0.0910759	1.37915	3.0892
2	1.55767	0.523276	0.198858	1.63316	3.00859
3	1.56476	0.551205	0.213047	1.6038	3.08931
4	1.69126	0.565262	0.0698163	1.77571	4.04036
5	1.55853	0.527446	0.011075	1.61754	3.65049
6	1.34446	0.495735	0.163291	1.39634	2.93292
7	1.4855	0.563982	0.095909	1.48269	3.5575
8	1.65732	0.571554	0.140501	1.63127	3.55948
9	1.49226	0.539854	0.169187	1.51568	4.63289
10	1.80011	0.698015	0.166575	1.83329	5.3144

Table S3. Projected mitral orifice area (MOA) of simulation cohort in cm^2 of single orifice (pre), orifices at anterior (ant) and posterior (post) commissures with clip in medial, central and lateral position and their sum, respectively. The area for anterior and posterior leaflets is provided in cm^2.

		medial			central			lateral			leaflet area	
case	pre	ant	post	sum	ant	post	sum	ant	post	sum	ant	post
1	6.65	2.52	0.16	2.68	1.30	0.70	2.00	0.19	2.28	2.47	7.78	8.21
2	7.63	1.87	0.70	2.57	0.92	1.48	2.40	0.44	2.96	3.40	12.41	12.22
3	5.85	1.62	0.17	1.79	0.58	0.95	1.53	0.32	1.69	2.01	11.61	12.75
4	10.79	3.67	0.17	3.84	1.96	1.36	3.32	0.56	2.65	3.21	15.05	14.09
5	7.24	1.84	0.06	1.90	0.88	1.07	1.95	0.16	2.91	3.07	12.93	12.38
6	6.40	2.18	0.25	2.43	0.93	0.74	1.67	0.27	1.42	1.69	9.70	7.86
7	5.90	2.95	0.20	3.15	1.87	0.41	2.28	0.92	1.08	2.00	10.09	12.15
8	7.31	2.18	1.03	3.21	0.83	1.71	2.54	0.46	2.55	3.01	14.13	13.19
9	7.38	2.10	0.41	2.51	0.94	1.20	2.14	0.13	1.99	2.12	10.85	11.31
10	9.83	2.30	0.83	3.13	1.21	1.92	3.13	0.41	3.18	3.59	16.44	15.50

the device virtually at each position. Streamline representations of the diastolic inflow at stress conditions are shown in Figure S3 and Figure S4 for all Patients and all simulated configurations.

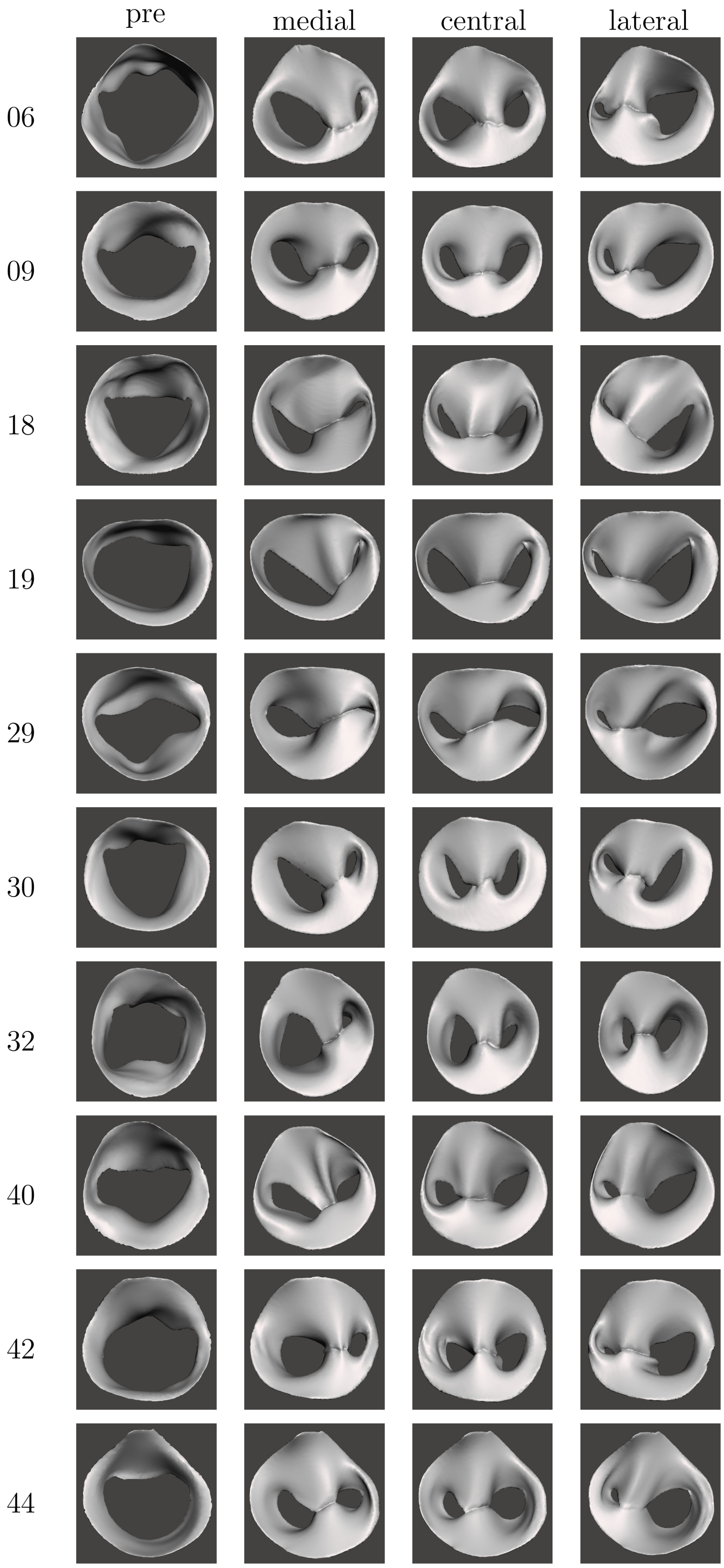

Figure S2: Mitral valve geometries pre-interventional (left row), and after virtual TEER with device in lateral, central and medial position.

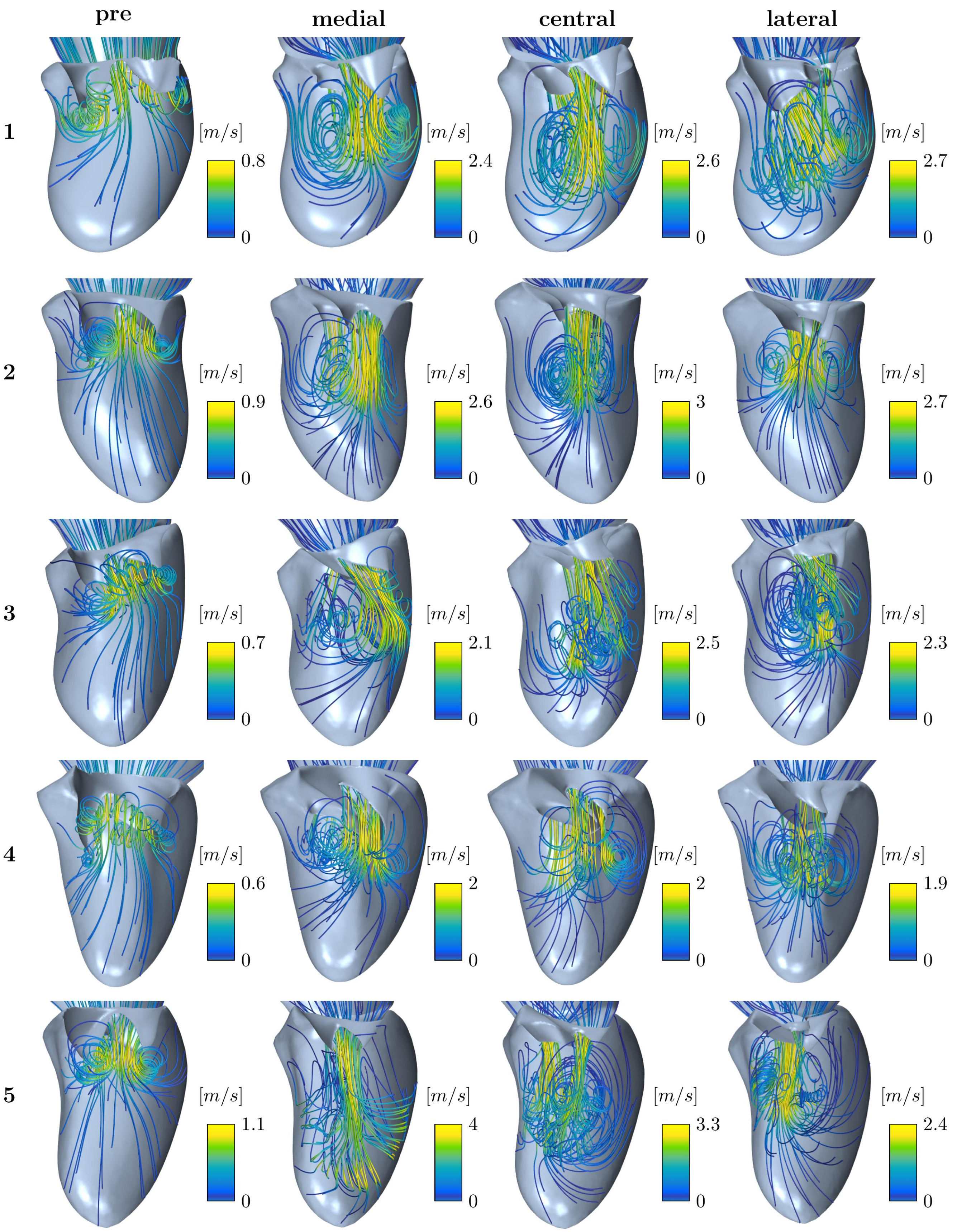

Figure S3: Streamlines of Patients 1 to 5 at early diastolic inflow simulations before and after virtual device implantation at medial, central and lateral position respectively.

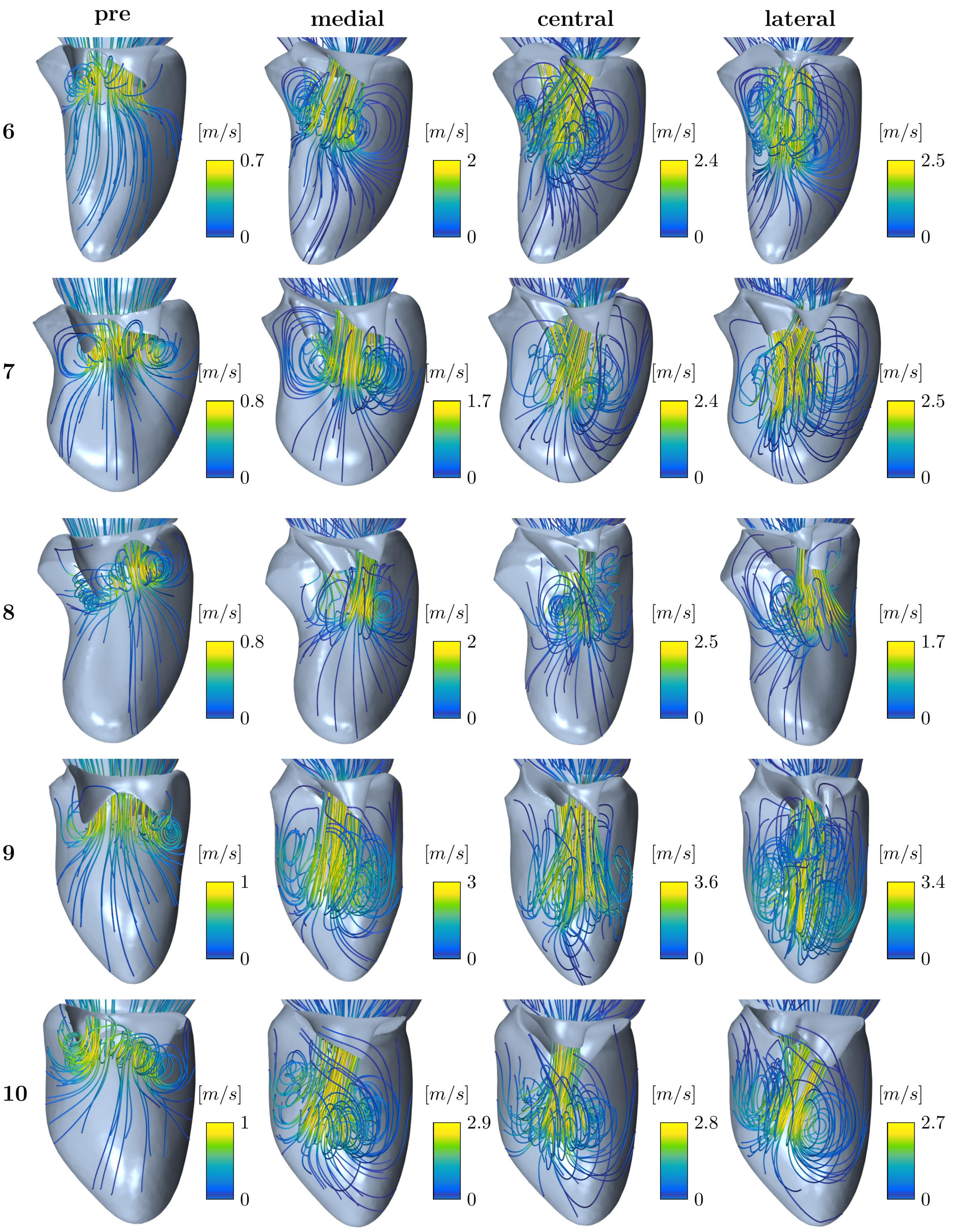

Figure S4: Streamlines of Patients 6 to 10 at early diastolic inflow simulations before and after virtual device implantation at medial, central and lateral position respectively.
